# Insights on diagnosis, prognosis and screening of renal cell carcinoma

**DOI:** 10.1590/S1516-31802002000600001

**Published:** 2002-11-01

## Introduction

Malignant tumors of the kidney account for about 1.9% of total world cancers, with about 150,000 new cases annu-ally.^1^ Renal parenchyma (renal cell) cancer represents around 80% of this total and nearly all of these cases are adenocarci-nomas.^2,3^ As a whole, it is difficult to obtain clear descriptive patterns of incidence and mortality from renal cell cancer, since population data from different regions are presented conjointly with pelvis and ureter cancers.^4^ In Latin America, the highest incidence rates of kidney cancer are observed in Montevideo, Uruguay (10.6 per 100,000 males) and in Porto Alegre, Brazil (10.2 per 100,000 males). The São Paulo City Cancer Registry has reported a lower rate (6.9 per 100,000 males), but this is higher than the rates reported in Belém and Goiânia.^4,5^ The male/female ratio of kidney cancer in São Paulo is 1.9:1. The incidence of renal cell cancer and the associated mortality have increased throughout the world^3^ over the last 50 years. In the city of São Paulo too, increasing incidence of kidney cancer and associated mortality was observed from 1969 to 1998, but the specific rates for renal cell cancer could not be shown.^5^

The etiology of kidney cancer has not been well established yet, except for renal pelvis and ureter cancers, the majority of which are related to tobacco smoking.^2^ Nevertheless, cigarette smoking has been associated with a moderate risk of renal cell cancer, according to several case-control and cohort studies. It is also suspected that some drugs, such as phenacetin and diuretics (hydrochlorothiazide and furosemide), may give rise to a risk of renal cell cancer. ^2^

### Results from international studies

In their comprehensive renal cell cancer survival analysis, Dall'Oglio et al.^6^ collected valuable data which allowed them to make some inferences regarding diagnosis and prognosis for this disease. In their methodological approach, the authors divided the study subjects into two groups, which were both submitted to the same surgical procedures: those with symptoms, for whom a 5-year survival rate of 80% was estimated, and another group, formed by subjects with incidental renal cell cancer diagnosis, with a 5-year survival rate of 100%.

Their results revealed survival rates higher than those observed in the United States. Data from the National Cancer Institute's Surveillance, Epidemiology, and End Results (SEER) Program have shown 5-year actuarial survival rates for renal cell cancer patients of 58% among white males and 59% among white females.^2^ The survival rates for black males and females were lower. The survival rates obtained by

Dall'Oglio et al.^6^ certainly cannot be assumed to represent the survival for the general population of the city of São Paulo because of the sources of their cases, i.e. two hospitals that treat patients from the highest social stratum of the population. Such a variable may have important implications on cancer survival, since this population has easier access to healthcare and those hospitals are renowned for their highly differentiated healthcare quality.

## Comments

Renal cell cancer survival has improved around the world,^2,3^ and is dependent upon earlier diagnosis, refinement in surgical techniques and advances in immunotherapy.^7-10^ The different rates observed between these two patient series may be attributable to the fact that32.2% of the symptomatic cases were classified in stages III or IV at the time of diagnosis. On the other hand, the majority of incidental cases (88.2%) were detected in stages I and II and none were classified in stage IV. These distinct survival rates observed between study groups are an illustrative demonstration of lead time bias, which is thought to exert an important influence on survival results following cancer screening.^11^ In the present study it can be postulated that incidental renal cell cancer cases seem to have better prognosis, either due to their lower degree of malignancy or their slower growth,^3^ and thus these characteristics may have artificially improved the survival of this group of cases.

However, no conclusive inference could be drawn from the results of the study by Dall'Oglio et al,^6^ in relation to the hypothesis to account for the fact that incidental renal cell cancer cases are a distinct clinical entity in comparison with symptomatic renal cell cancer cases. This was because no investigations were conducted on molecular biomarkers or differences in survival according to tumor cell type between the groups. Furthermore, the subtype seemed to be an independent prognostic variable for renal cell cancer survival.^8,9^

As emphasized by the authors of that study, the increase in renal cell cancer diagnosis at its early stages is likely to have been a consequence of advanced renal imaging technology. In fact, this has been considered to be the major explanation for the increase in renal cell cancer incidence rates in many regions over recent decades. Dissemination of the use of this new imaging technology may have resulted in a nonsystematic partial screening of the population for early renal cell can- cer.^3,12^ Nonetheless, there has also been an increase in advanced cases around the world, including those with regional extension and distant metastases. The final consequence of this has been increased mortality due to the disease.^2,3^

## Conclusion

On the basis of this review study, the main issue to be pointed out is that the discovery of an incidental renal cell cancer case presupposes prompt measures for treating the disease. As the authors stated, this will improve patients’ prognosis and quality of life. Meanwhile, while at the individual level there is a clear benefit from early renal cell cancer diagnosis and treatment, obviously nothing could be concluded about the benefits of public screening programs for renal cell cancer.

There is no evidence for the effectiveness of the screening approach to control renal cell cancer at the level of populations.^13^
